# Acquired Vitamin K Deficiency as Unusual Cause of Bleeding Tendency in Adults: A Case Report of a Nonhospitalized Student Presenting with Severe Menorrhagia

**DOI:** 10.1155/2017/4239148

**Published:** 2017-08-27

**Authors:** Omid Reza Zekavat, Gholamreza Fathpour, Sezaneh Haghpanah, Seyed Javad Dehghani, Maryam Zekavat, Nader Shakibazad

**Affiliations:** ^1^Hematology Research Center, Shiraz University of Medical Sciences, Shiraz, Iran; ^2^Department of Biological Engineering, Massachusetts Institutes of Technology, Cambridge, MA, USA

## Abstract

We report a rare case of acquired vitamin K deficiency presenting with severe menorrhagia and without any gynecological problem. Partial thromboplastin time (59.2 seconds) and prothrombin time (33.1 seconds, INR: 5.97) were considerably prolonged in laboratory evaluations. A complete coagulation factor assay test was performed for the patient: factor IX, 24%; factor II, 41%; factor VII, 3%; and factor X, 52%. She had been taking many high-energy drinks and she had inadequate dietary intake for the past 6 months. Given that she had vitamin K deficiency (VKD), a course of vitamin K therapy was started for her in the hospital. This case showed the potential for menorrhagia due to VKD with use of high-energy drinks and the value of a complete and detailed history in early diagnosis.

## 1. Introduction

Menorrhagia is a very common clinical condition reported in women of reproductive age. More than half of women with a normal gynecological evaluation will have laboratory abnormalities indicating hemostasis [1]. Menorrhagia can be the first manifestation of a bleeding disorder [2]. The estimated incidence of bleeding disorders as a cause of menorrhagia can be as high as 10–20% [1]. Coagulopathies are not usually suspected in the etiology of menorrhagia and surgical interventions are done without investigation for coagulopathies [3]. Normal individual with acquired vitamin K deficiency (VKD) as a cause of menorrhagia is rare, because of small daily requirement of vitamin K [4].

It is essential for physicians to perform logical clinical and laboratory assessments for an underlying bleeding disorder after gynecological causes are ruled out. Here, we report a rare case of acquired transient VKD as a cause of menorrhagia that is easily preventable in nonhospitalized patient.

## 2. Case

A 23-year-old healthy female consulted with hematologist for the condition of menorrhagia (heavy menstrual bleeding) indicated by the fact that she had to change her pad every 3-4 hours for duration of 3 months. She was teacher and student of master degree of psychiatry. She had been working for long periods of time without any rest for a year. For a period of 4 months prior to seeking medical advice, she had taken an excess of acetaminophen due to body pain. She did not have any past medical history or family history of bleeding tendency or a history of drugs (warfarin or antibiotic). On physical examination, she was only pale. Also, she had no gynecological problem or history of weight loss.

She was evaluated for possible cause of bleeding tendency. Her liver function tests, collagen-vascular disease tests, and thyroid function tests were normal (Table 1). Celiac panel tests also were normal.

In her laboratory work-up, hemoglobin was low (10.9 g/dl) and leukocyte count (5.4 × 10^3^ cell/*µ*L) and platelet count (268 × 10^3^ cell/*µ*L) were normal (Sysmex KX-21N™); but in evaluation of the coagulation system, bleeding time was prolonged (9 minutes, 11 seconds) (IVY method). Also partial thromboplastin time (PTT: 59.2 seconds) and prothrombin time (PT) were significantly prolonged (PT: 33.1 seconds, INR: 5.97) which were corrected in a mixing test without incubation time. Von Willebrand factor (vWF) antigen, vWF activity, and fibrinogen level were normal, and only mild factor X deficiency (8%) had been detected.

Due to iron deficiency signs and symptoms and ferritin 33.3 ng/mL, a course of iron therapy (4 mg/kg/day) with folic acid (1 mg per day) was recommended. Moreover, acetaminophen was discontinued.

In clinical and paraclinical follow-up, her signs and symptoms became better two weeks after iron therapy and bleeding time became normal (4 minutes and 15 seconds, IVY method), but her bleeding tendency continued. Because new history of acute severe dizziness caused her to have trouble walking, standing, or losing her balance, she was admitted to hospital for better evaluation. PTT was slightly prolonged (38.5 seconds) but PT was considerably high (21.2 seconds, INR: 2.51). As she had normal liver function tests, overdose of acetaminophen or liver disease was ruled out. A complete coagulation factor assay test was performed for her, and the results were as follows: factor IX, 24%; factor II, 41%; factor VII, 3%; and factor X, 52%. No inhibitors were detected. Again a detailed history was taken. She stated that for the past 6 months she had been taking many high-energy drinks and she had inadequate dietary intake. Given that she had VKD, a course of vitamin K therapy was started for her in the hospital. Coagulation study results became normal after 72 hours of therapy, and she was discharged from hospital with the diagnosis of VKD. Factor assay test became normal after one week of vitamin K therapy, with a dosage of 5 mg orally twice per day. Coagulation tests were repeated and the results included normal PTT, normal PT (INR: 1.01), and factor IX, 102%; factor II, 99%; factor VII, 97%; and factor X, 115% (IL ACL 7000 Coagulation Analyzer). The patient was on regular follow-up with the normal physical exam and without complaining of bleeding tendency.

## 3. Discussion

Heavy menstrual bleeding that occurs at regular intervals or at the onset of menses is often related to a bleeding diathesis and less commonly to systemic illness or structural lesions.

Given inherited bleeding disorders are found in 5 to 28% of hospitalized adolescent females with heavy menstrual bleeding; thus, these disorders should be considered in differential diagnosis [5, 6]. Vitamin K acts as a cofactor in *γ*-carboxylation of factors II, VII, IX, and X. VKD results in reduced biological activity of these factors presenting with increases in PT or aPTT, which are corrected by vitamin K therapy [7]. Because factor VII has the shortest half-life, PT becomes prolonged first. Serum concentration of phylloquinone (vitamin K1), vitamin K1 2, 3-Epoxide (K1 O), and PIVKA-II (under carboxylated factor II) can be measured, but usually the diagnosis is clinical and by exclusion [8]. These specific vitamin K concentrations were not measured in our patient due to unavailability of the test in our research center.

Humans do not maintain large stores of vitamin K and anything that interrupts the supply from diet or gut bacteria can result in a deficiency [9]. An adult daily intake of about 100 micrograms of phylloquinone is recommended for the maintenance of hemostasis [10]. There are two kinds of combined deficiency of the vitamin K-dependent clotting factors. The first, hereditary combined deficiency of the vitamin K-dependent clotting factors is an inherited enzyme deficiency involving vitamin K metabolism that is an extremely rare autosomal recessive disorder in adults and is present in fewer than 30 kindred worldwide [5]. The second one is due to nutritional vitamin k deficiency commonly seen in neonate as hemorrhagic disease of newborns [11]. Acquired VKD in nonhospitalized adults manifesting as overt spontaneous bleeding especially menorrhagia is a rare phenomenon. Although VKD theoretically could occur in these patients, the very small daily requirement of vitamin K means it does not happen commonly [12].

Secondary VKD can also occur in patients undergoing major surgery or in those treated with long-term parenteral nutrition and broad-spectrum antibiotic therapy [13]. Secondary VKD is caused by the inhibition of the hepatic vitamin K epoxide reductase enzyme, resulting in impaired vitamin K recycling. Extremely high doses of vitamins E and A can cause VKD [14]. Secondary VKD can also occur in bulimia, those on stringent diets, and those taking anticoagulants [15].

Our patient was diagnosed with secondary VKD, resulting from inadequate nutritional intake (with a diet containing mostly energy drinks) over a period of several months, in addition to excessive acetaminophen intake. Energy drinks (consumed by our patients) contain amounts of vitamins A and E without vitamin K, which can explain high daily intake relating to VKD. Physicians should keep acquired VKD in mind as a differential diagnosis of bleeding diathesis in nonhospitalized patients, especially in patients with inadequate nutritional intake. This case showed the potential for menorrhagia due to secondary VKD with use of high-energy drinks and the value of a complete and detailed history in early diagnosis.

Recognition of VKD is critical to make appropriate treatment and prevention of treatment failure, because underlying hemostatic disorder may lead to continued menorrhagia and diminished quality of life due to unnecessary gynecologic surgical interventions. We also suggest further research on contents of commercial energy drinks which affect acquired VKD.

## Figures and Tables

**Table 1:** Laboratory findings in our case report.

	Result	Unit
*Liver function tests*		
AST	17	U/L
ALT	15	U/L
Total bilirubin	0.48	Mg/dL
Direct bilirubin	0.17	Mg/dL
Albumin	4	g/dL
Alkaline phosphatase	110	U/L

*Collagen-vascular disease tests*		
ANA	Negative	
anti-dsDNA	Negative	
ACLA	Negative	
APLA	Negative	

*Thyroid function tests*		
TSH	1.97	*µ*IU/ml
T4	102.70	nmol/L
T3	1.27	nmol/L

AST: aspartate aminotransferase, ALT: alanine aminotransferase, ANA: antinuclear antibody, anti-dsDNA: anti-double-stranded DNA, ACLA: anticardiolipin antibodies, APLA: antiphospholipid antibodies, TSH: thyroid-stimulating hormone, and T4: thyroxine.
